# Characterization and Functional Analysis of RhHsfA7, a Heat Stress Transcription Factor in Roses (*Rosa hybrid* ‘Samantha’)

**DOI:** 10.3390/plants14081155

**Published:** 2025-04-08

**Authors:** Yaqi Sun, Sudan Li, Xiang Wu, Jiao Zhu, Fei Dong, Zhaoshun Pei, Zhenguo Li, Shanxing Zhao, Chengpeng Wang

**Affiliations:** 1 Shandong Engineering Research Center of Ecological Horticultural Plant Breeding, Institute of Leisure Agriculture, Shandong Academy of Agricultural Sciences, Jinan 250100, China; sunyaqi123456@sina.com (Y.S.); 15515752749@163.com (S.L.); wwuxiang5@163.com (X.W.); mmzhujiao520@163.com (J.Z.); dongfei860117@163.com (F.D.); qp1587804448@163.com (Z.P.); 15689033552@163.com (Z.L.); zsx5678@163.com (S.Z.); 2 Shandong Haoyu Horticulture Technology Limited Liability Company, Rizhao 276800, China

**Keywords:** rose, RhHsfA7, heat tolerance, ornamental plants

## Abstract

Heat stress transcription factors (Hsfs) are crucial transcription factors (TFs) in plants, playing pivotal roles in responding to abiotic stresses. However, their specific functions in regulating heat stress responses in roses are not yet fully elucidated. Here, we cloned an Hsf gene, *RhHsfA7*, from the rose variety *Rosa hybrid* ‘Samantha’. This gene contains a coding sequence (CDS) of 1086 bp, encoding 361 amino acids. The RhHsfA7 protein has a molecular weight of 41.21 kDa, an isoelectric point of 5.41, and no signal peptide or transmembrane structure. Phylogenetic analyses revealed that *RhHsfA7* is most closely related to *AtHsfA7a*, *AtHsfA7b*, and *AtHsfA6b* in *Arabidopsis thaliana*, and is phylogenetically closer to *Rosaceae* species compared to other species. The RhHsfA7 protein possesses conserved domains, including an oligomerization domain (OD), a nuclear localization signal (NLS), a DNA-binding domain (DBD), and a nuclear export signal (NES), as well as the HsfA subfamily-specific transcriptional activation domain (AHA). RhHsfA7 was localized in the nucleus and exhibited transcriptional activation activity. Expression analysis revealed that *RhHsfA7* was highly expressed in roots and leaves, and its expression was heat-specific. In rose leaves, through silencing and transient overexpression experiments, we discovered that silencing *RhHsfA7* resulted in heat sensitivity, whereas transient overexpression of *RhHsfA7* increased heat tolerance. Collectively, our findings suggest that *RhHsfA7* positively regulates tolerance to heat stress in roses.

## 1. Introduction

The rose (*Rosa hybrida*) is a perennial evergreen shrub belonging to the Rosaceae family. Their long flowering period, pleasant fragrance, and rich floral colors make roses extremely ornamental, and they rank first in the world fresh-cut-flower market [1,2]. Nevertheless, roses prefer a humid and warm living environment with ample light [3]. High temperatures in summer can cause the abnormal development of roses, such as bent peduncles [4], leaf yellowing and withering [3], decreased flower size, and other physiological diseases [5], which seriously affect their ornamental quality and economic value. As global warming leads to frequent high temperatures in summer, rose production faces the severe challenge of unforeseeable high temperatures. Therefore, investigating genes involved in the rose heat stress response is crucial. Meanwhile, multiple defense strategies, including stress sensing, signal transmission, and transcriptional regulation, have been developed by plants for adaptation to abiotic stresses [6]. Previously, several transcription factors (TFs) like DREB, HSF, MYB, and bZIP have been shown to act as key regulators in responding to biotic or abiotic stresses in certain horticultural plants [7,8].

Heat stress TFs (Hsfs) are the most critical plant TFs [9]. Hsfs respond to heat stress and other abiotic stresses like drought, salt, and cold through combining with heat stress elements (HSEs) and activating heat shock protein (Hsp) expression [10,11]. Though with distinct functions, plant Hsfs share highly similar patterned structures comprising an oligomerization domain (OD), a nuclear localization signal (NLS), and a DNA-binding domain (DBD); some contain a nuclear export signal (NES) as well [12]. They are classified into classes A, B, and C based on OD characteristics [13], with extensive research attention focused on class-A Hsfs (HsfAs) due to their critical roles in plants’ heat stress response. HsfAs typically contain several acidic motifs (AHAs) at their C-terminus, which act as transcriptional activators [13]. HsfAs have been reported to widely participate in responding to heat and other stresses in plants. For example, *HSFA6b* serves as a positive regulator in *Arabidopsis* for ABA-mediated drought and salinity resistance and is necessary for establishing thermotolerance [14]. Knockout mutants of *HsfA7a* and *HsfA7b* in *Arabidopsis* show reduced heat tolerance compared to the wild type (WT) [15,16]. In tomato, HsfA7 regulates the mild-to-severe heat stress response transition by modulating *HsfA1a* activity [17]. Overexpressing *Apium graveolens AgHSFa6-1* in *Arabidopsis* increases heat tolerance by promoting ROS scavenging, osmoregulation, and the upregulation of heat-resistance-related gene expression [18]. *CaHSFA6a* confers heat tolerance in *Capsicum annuum* [19]. *LlHsfA2* from *Lilium longiflorum* is induced by H_2_O_2_ and heat stress [20] and regulates heat responses by interacting with LACTIN [21]. The heterologous expression of *Camellia sinensis CsHsfA2* in transgenic yeast confers heat tolerance [22]. Hsfs are critical regulators of thermotolerance in plants, yet their functional characterization in *Rosa* remains limited. Recent genome-wide studies identified 19 Hsf genes in *Rosa chinensis*, classified into the A, B, and C subfamilies, with specific members like *RcHsf17* and *RhHsf24* showing strong heat-inducible expression and roles in enhancing thermotolerance through ROS detoxification and stress signaling modulation [23,24,25]. Despite these advances, research on *Rosa* Hsfs is still sparse compared to that on model plants like *Arabidopsis*. 

In this research, an Hsf gene, *RhHsfA7*, was isolated from *R. hybrida* ‘Samantha’. Phylogenetic relationships, conserved domains, subcellular localization, transcriptional activity, expression patterns in different organs, and the response to stress were all analyzed. According to these results and previous research, it is inferred that this gene plays a role in plants’ responses to high-temperature stress. In order to validate this hypothesis, the gene was silenced or overexpressed in rose leaves to characterize its function in rose thermotolerance. The results demonstrate that *RhHsfA7* can enhance tolerance to heat stress in roses. This study provides a theoretical foundation for further examinations of the molecular mechanism of *RhHsfA7* in heat stress responses in roses.

## 2. Results

### 2.1. Cloning and Bioinformatics Analyses of RhHsfA7

**Figure 1:**
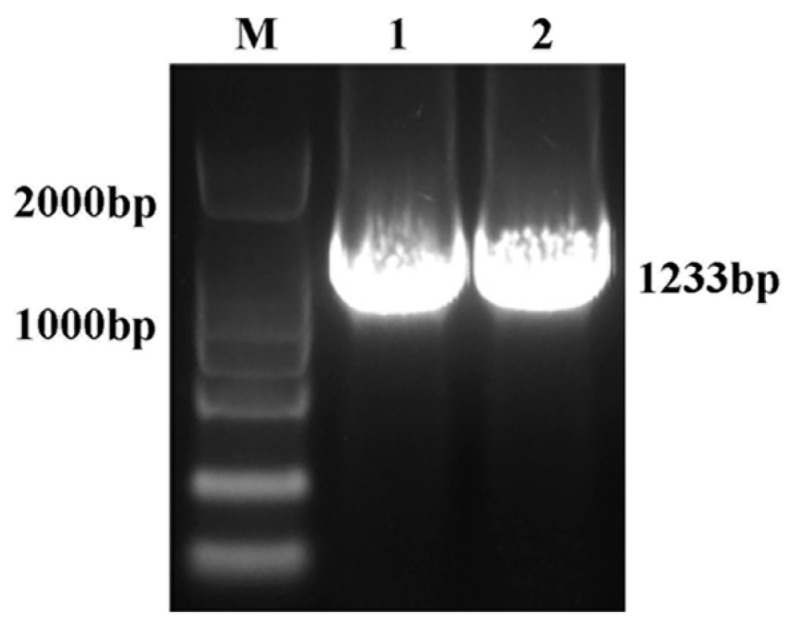
Gel electrophoresis results of *RhHsfA7* cloning. M: BM2000 DNA Marker; Lanes 1 and 2: amplification products of *RhHsfA7* gene.

**Figure 2:**
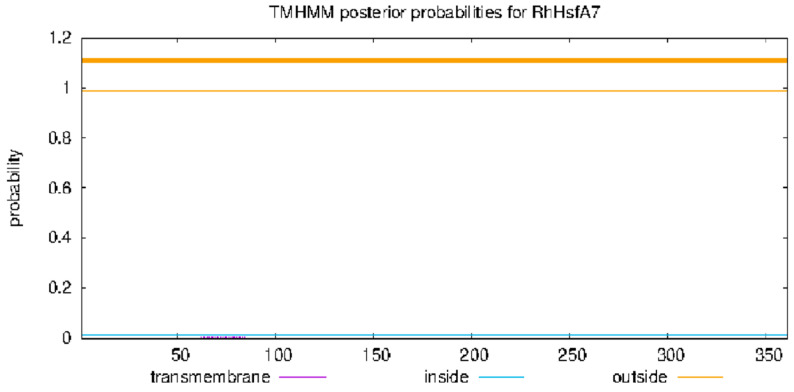
Transmembrane domain prediction of RhHsfA7 protein.

**Figure 3:**
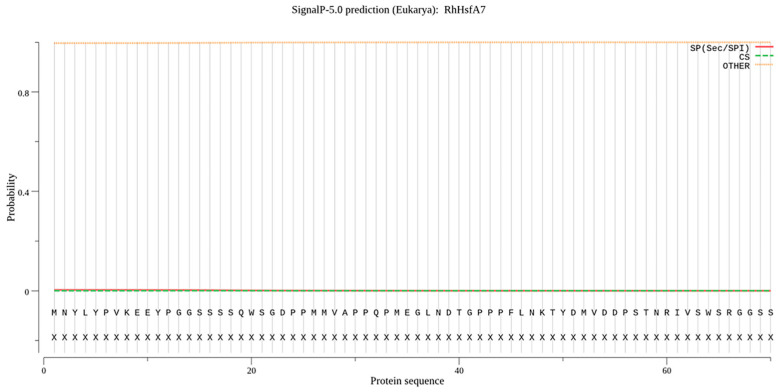
Signal peptide prediction of RhHsfA7 protein.

The 1086 bp coding sequence of *RhHsfA7* was cloned from *R. hybrida* ‘Samantha’ (Figure 1). Subsequently, the protein was analyzed and found to consist of 361 amino acids. RhHsfA7 has an isoelectric point of 5.41 and a molecular mass of 41.21 kDa, indicating its hydrophilicity and stability. The predictive results for the transmembrane structure (Figure 2) and the signal peptide (Figure 3) revealed the absence of both a transmembrane domain and a signal peptide in RhHsfA7, confirming its status as a non-secreted protein.

**Figure 4:**
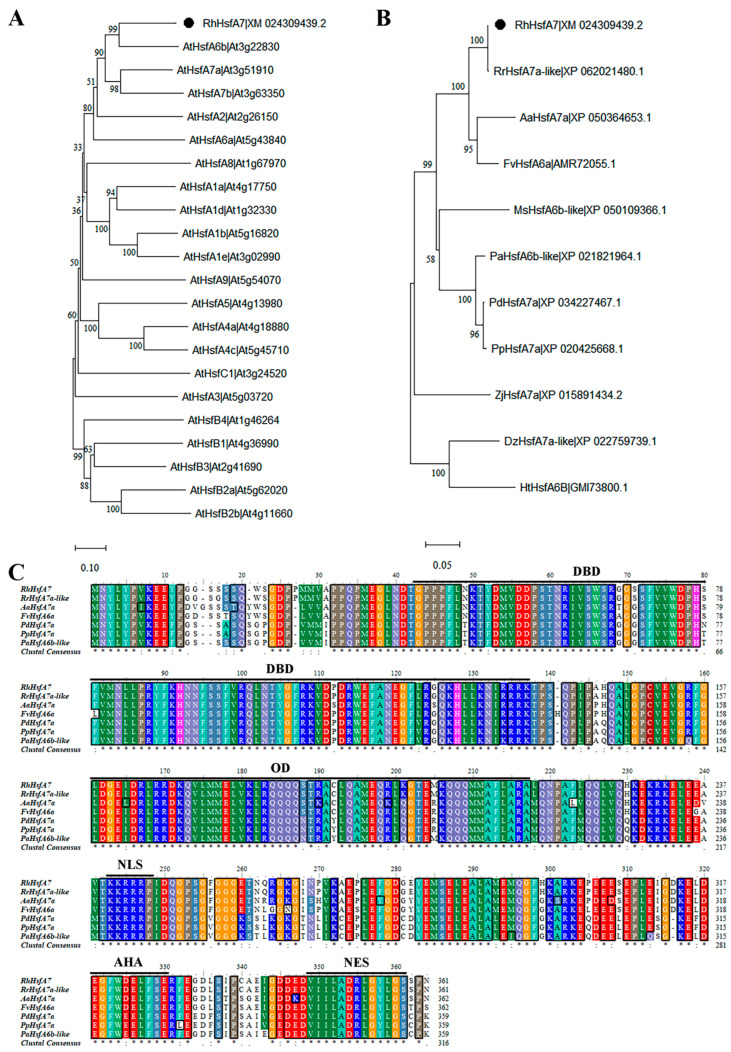
Phylogenetic analysis of and homologous evolutionary relationship between RhHsfA7 and Hsf proteins of other species. Phylogenetic trees of RhHsfA7 and Hsf proteins from (**A**) *Arabidopsis* and (**B**) ten other species were constructed. The target protein, RhHsfA7, is marked by black circles. (**C**) Multiple sequence alignment analysis between RhHsfA7 and six other Hsf proteins, highlighting the typical OD, DBD, NLS, NES, and AHA domains represented by black solid lines.

Through a phylogenetic analysis of all *Arabidopsis* Hsfs, *RhHsfA7* was found to be most closely related to *AtHsfA7a*, *AtHsfA7b*, and *AtHsfA6b*, suggesting that they may share similar biological functions (Figure 4A). In addition, through sequence alignment, Hsfs with high sequence similarity to *RhHsfA7* were selected. A phylogenetic tree analysis of these eleven Hsfs from different species revealed that *RhHsfA7* showed a higher degree of similarity to members of the Rosaceae family, such as *Rosa rugosa*, *Argentina anserina*, and *Fragaria vesca* (Figure 4B). Furthermore, through amino acid sequence alignment with Hsf proteins from six different species, it was observed that the RhHsfA7 protein, similarly to other Hsf proteins, possesses conserved OD, DBD, NLS, and NES domains, along with a special domain of the HsfA subfamily, namely, the AHA domain (Figure 4C).

### 2.2. Structural Prediction of RhHsfA7 Protein

**Figure 5:**
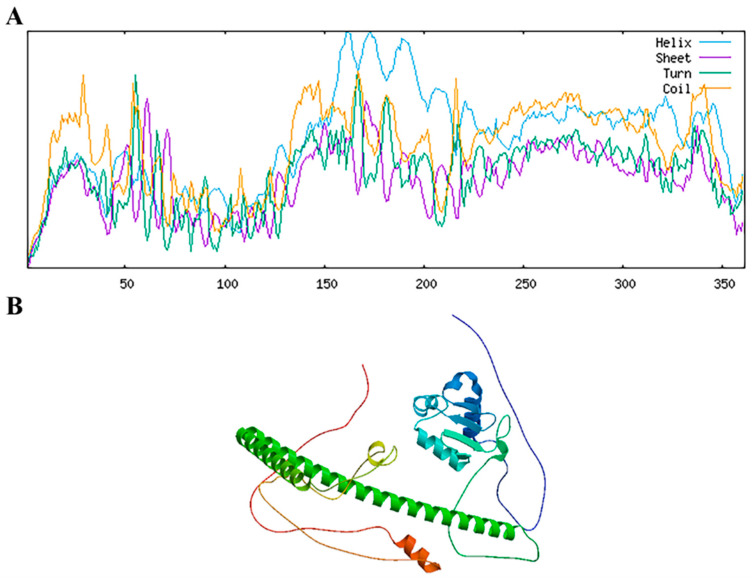
Predicted structure of RhHsfA7 protein. (**A**) Secondary structure and (**B**) tertiary structure of RhHsfA7 protein.

The secondary structure analysis of the RhHsfA7 protein showed that it contains abundant alpha helices and random coils (Figure 5A). The tertiary structure of the RhHsfA7 protein was obtained using the SWISS-MODEL tool, which was consistent with the predicted secondary structure and contained a large number of α helices and aperiodic coils (Figure 5B).

### 2.3. Subcellular Localization and Transactivation Activity of RhHsfA7 Protein

**Figure 6:**
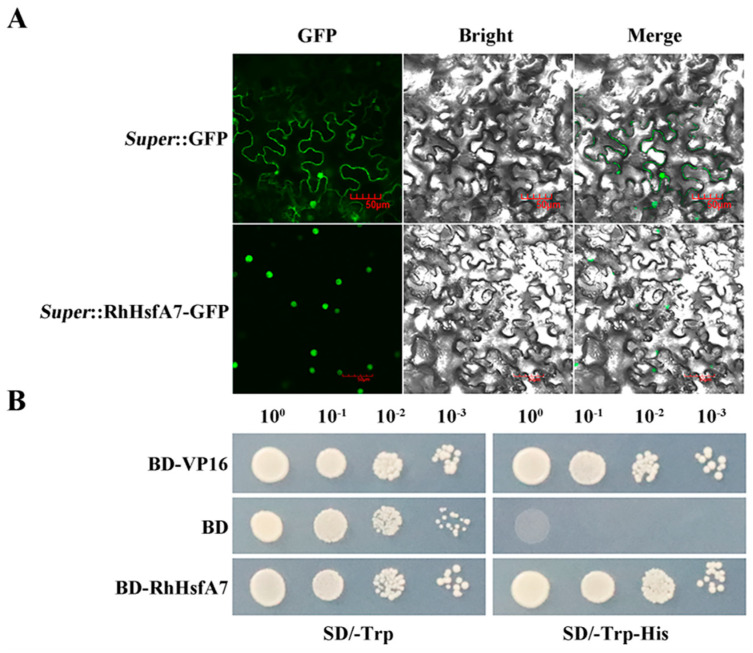
Subcellular localization and transcriptional activity assay of RhHsfA7. (**A**) Subcellular localization of RhHsfA7-GFP in tobacco cells. Scale bars: 50 µM. GFP—green fluorescent protein. (**B**) Transcriptional activity assay of RhHsfA7 in yeast cells. Transformants carrying pGBKT7 (BD), positive control (BD-VP16), and BD-RhHsfA7 were spared on SD/-Trp-His and SD/-Trp media and cultured for 3 days. The 10^0^, 10^−1^, 10^−2^, and 10^−3^ numbers represent dilution factors.

To determine the subcellular localization of RhHsfA7, the *Super*:RhHsfA7-GFP fusion protein was transiently expressed in tobacco leaves. Observation by microscopy displayed that *Super*:RhHsfA7-GFP only exhibited GFP fluorescence in the nucleus, while Super:GFP alone exhibited fluorescence in the entire cell (Figure 6A). This indicated that the RhHsfA7 protein was localized in the nucleus.

For the detection of transcriptional activity, the recombinant pGBKT7-RhHsfA7 (BD-RhHsfA7) vector was constructed and transformed into yeast cells. As depicted in Figure 6B, all transformants grew well on the tryptophan (Trp)-free synthetic dropout (SD) medium. In addition, normal growth of BD-RhHsfA7 and the positive-control yeast cells was found on SD plates lacking Trp (SD/−Trp−His) or histidine (His), suggesting the presence of transcriptional activation activity in RhHsfA7. Taken together, these results indicate that RhHsfA7 is a nuclear-localized transcription activator, demonstrating its transcription factor characteristics.

### 2.4. Expression Analyses of RhHsfA7 in R. hybrida

**Figure 7:**
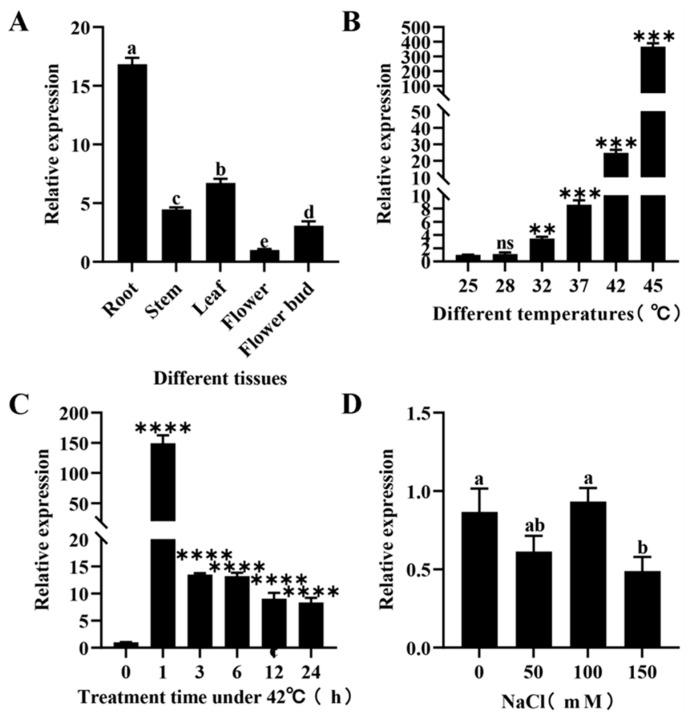
The patterns of *RhHsfA7* expression. (**A**) Expression profiles of *RhHsfA7* in different tissues. (**B**,**C**) Expression profiles of *RhHsfA7* under heat stress. (**D**) The expression of *RhHsfA7* under salt stress. (**B**,**C**) Data were compared using Student’s t-tests (ns *p* > 0.05, ** *p* ≤ 0.01, *** *p* ≤ 0.001, **** *p* ≤ 0.0001). (**A**,**D**) Data were compared using one-way analyses of variance with a significance level of 0.05, with significant differences indicated by different lowercase letters (*p* < 0.05).

To investigate the tissue-specific expression patterns of *RhHsfA7*, the expression levels of *RhHsfA7* in leaves, stems, roots, flowers, and flower buds of *R. hybrida* were tested with quantitative reverse transcription PCR (qRT-PCR). The results showed that *RhHsfA7* has various expression levels in different tissues, with levels being higher in roots and leaves, while being lower in stems, flower buds, and flowers (Figure 7A). Next, we analyzed the expression pattern of *RhHsfA7* in leaves subjected to heat stress or NaCl treatment. As shown in Figure 7B, the expression of *RhHsfA7* gradually increased with increasing temperature, reaching the highest level at 45 °C. In addition, *RhHsfA7* expression notably increased at 42 °C for 1 h, and then reduced, while remaining significantly higher than the control (Figure 7C). Furthermore, *RhHsfA7* expression was notably reduced only at a concentration of 150 mM NaCl compared to the control (Figure 7D). These findings indicate that the *RhHsfA7* gene is induced by heat stress but not by salt stress.

### 2.5. RhHsfA7 Silencing Decreased Heat Tolerance in Rose

**Figure 8:**
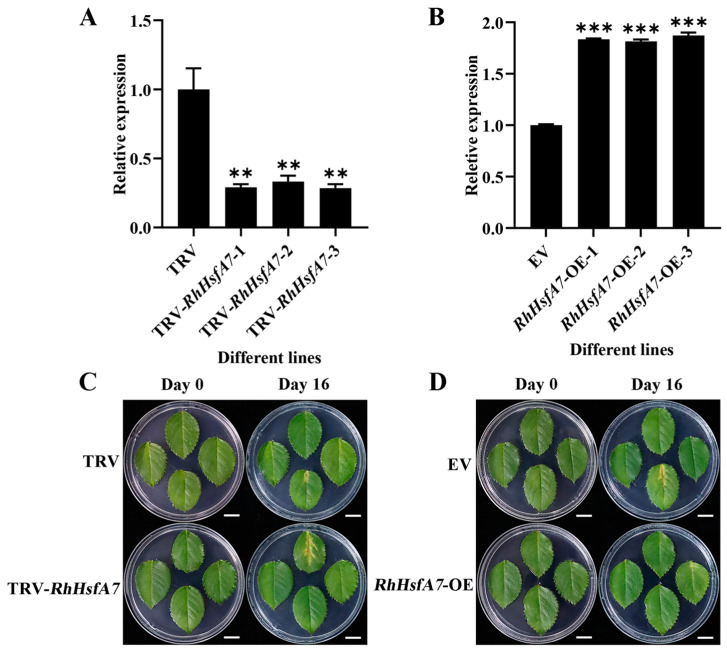
RhHsfA7 positively regulates heat tolerance in rose. (**A**) The relative expression of *RhHsfA7* in TRV-*RhHsfA7* and TRV control leaves. (**B**) The relative expression of *RhHsfA7* in EV and *RhHsfA7*-OE rose leaves. (**C**) Phenotype of TRV-*RhHsfA7* and TRV control leaves after heat stress. (**D**) Phenotype of EV and *RhHsfA7*-OE rose leaves after heat stress. Scale bar: 1 cm. (**A**,**B**) Data were compared using Student’s *t*-tests (** *p* ≤ 0.01, *** *p* ≤ 0.001).

To assess the function of *RhHsfA7* under heat stress, the *RhHsfA7* gene was silenced in rose leaves using a VIGS approach [26]. *RhHsfA7* was silenced in rose leaves, which were then subjected to heat stress (42 °C) treatment for 12 h. Compared to the tobacco rattle virus (TRV) control, the TRV-*RhHsfA7* leaves showed 66% lower *RhHsfA7* expression levels (Figure 8A). No significant phenotypes were observed in either TRV or TRV-*RhHsfA7* leaves before heat stress. In contrast, after heat stress, TRV-*RhHsfA7* leaves exhibited more severe yellowing and browning on the 16th day than the TRV control (Figure 8C). The results demonstrate that silencing *RhHsfA7* in roses leads to a decrease in heat tolerance.

### 2.6. Overexpressing RhHsfA7 Improved Heat Tolerance in Rose

Next, *RhHsfA7* was transiently overexpressed in rose leaves, generating *RhHsfA7*-overexpressing (*RhHsfA7*-OE) leaves. Compared to the empty vector (EV) control, the *RhHsfA7*-OE leaves exhibited a 1.8-fold higher *RhHsfA7* expression level (Figure 8B). Before heat treatment, we found no phenotypic differences between EV and *RhHsfA7*-OE leaves. However, after a 42 °C treatment for 12 h, the EV leaves turned yellow and brown on the 16th day, while *RhHsfA7*-OE leaves exhibited a milder degree of yellowing (Figure 8D). The findings indicate that overexpressing *RhHsfA7* in roses results in enhanced heat tolerance.

## 3. Discussion

High temperatures are harmful to the quality and yield of roses, affecting the development of the rose industry. When faced with unfavorable factors during growth and development, plants demonstrate complex regulatory mechanisms that they have developed. The regulation of related genes by TFs plays a crucial role in these mechanisms; therefore, exploring candidate genes for heat tolerance is a key strategy in rose breeding. Hsfs are TFs that widely exist in all organisms and play core roles during plant development, growth, and responses to abiotic stresses [27,28]. However, research on the functions of the rose Hsf family in response to stress is still limited. Therefore, we cloned an Hsf gene from roses and studied its protein characteristics, transcription factor properties, gene expression patterns, and function.

Based on structural analysis, RhHsfA7 was found to have conserved DBD, OD, NLS, AHA, and NES domains, classifying it into the HsfA subfamily. This classification provides a foundation for investigating its function. *Arabidopsis* AtHSFA7b is located in the nucleus and has transcriptional activation activity [16], which is consistent with the results of this study, indicating that RhHsfA7 can function as a transcriptional activator. Phylogenetic analysis showed that *RhHsfA7* is homologous to AtHsfA7a, AtHsfA7b, and AtHsfA6b, all of which are involved in the response to heat stress [14,15,16]. Therefore, *RhHsfA7* may play a role in the heat stress response in roses, offering a basis for further research.

Exploring the expression patterns of genes can help in understanding their biological functions [29]. Here, the expression of the *RhHsfA7* gene in five tissues and under stress conditions was investigated. *RhHsfA7* was expressed at the highest levels in roots, followed by leaves, stems, flower buds, and then flowers. This is consistent with tea plant *CsHsfA7* [22] and cannabis *CsHsf10* [30] having the highest expression levels in roots. However, *RcHsf17* was highly expressed in rose leaves, followed by flowers and roots [23]. These findings demonstrate that Hsfs exhibit tissue-specific expression patterns.

Next, we investigated the expression level of *RhHsfA7* in leaves under stress treatment. *RhHsfA7* expression gradually increased with rising temperature, unlike that of Rhododendron *RsHsf3* (*RsHsfA7* subclass), which gradually decreased with increasing temperature [31]. This might be due to differences between species or genes. During heat treatment at 42 °C, the expression of *RhHsfA7* was significantly upregulated and gradually decreased after one hour, yet it remained higher than the control. Its expression pattern was similar to that of strawberry *FvHsfA6a* and Hypericum *HpHSF-23* [32,33]. The findings suggest the potential involvement of RhHsfA7 in regulating heat tolerance in roses. However, the expression level of *RhHsfA7* did not show a regular pattern of change under salt stress, indicating that RhHsfA7 may not be involved in regulating salt tolerance in roses.

We performed further functional validation studies on this gene, and demonstrated the positive role of *RhHsfA7*, which is homologous to tomato *HSFA7*, in heat stress tolerance. Tomato *HSFA7* is induced by heat stress, affects phenotypic changes, and enhances the plant’s heat stress tolerance [34]. Consistently, our results showed that silencing *RhHsfA7* reduced the heat tolerance of leaves, while overexpressing *RhHsfA7* significantly enhanced heat tolerance. This reveals that RhHsfA7 serves as a positive regulator of rose heat tolerance, laying a foundation for future studies on the regulatory mechanism of RhHsfA7.

## 4. Materials and Methods

### 4.1. Plant Material and Treatments

*Rosa hybrida* ‘Samantha’ was used as the experimental material. Prior to the study, leaves were spread flat on 0.4% (*m*/*v*) agar culture plates. For heat stress treatment, leaves cultured on water agar plates were exposed to different temperatures (25, 28, 32, 37, 42, and 45 °C) for 3 h or 42 °C for various durations (0, 1, 3, 6, 12, and 24 h) in incubators. For salt stress treatment, the leaves were transferred to water agar plates containing 0, 50, 100, and 150 mM NaCl, respectively, for 2 d. For tissue expression analyses, root, stem, leaf, flower, and flower bud samples were collected. Each sample type was represented in biological triplicates. Samples were rapidly frozen in liquid nitrogen and stored at −80 °C.

### 4.2. Cloning and Sequence Analysis of RhHsfA7

We extracted RNA from roses using the SPARKeasy Plant RNA Kit (SparkJade Biotech, China), followed by reverse transcription into cDNA using the SPARKscipt II RT Plus Kit (SparkJade Biotech, China). The coding sequence (CDS) of the *RhHsfA7* (XM_024309439.2) gene was amplified by PCR using the primers shown in Supplementary Materials Table S1.

For sequence analysis, the amino acid sequence of *RhHsfA7* was translated by using the Expasy-Translate tool (https://web.expasy.org/translate/) (accessed on 23 March 2025). Based on the protein sequence of RhHsfA7, the BLAST + 2.16.0 function on the NCBI website was used to collect 10 other highly homologous Hsf proteins, namely, RrHsfA7a-like (*Rosa rugosa*, XP_062021480.1), AaHsfA7a (*Argentina anserina*, XP_050364653.1), FvHsfA6a (*Fragaria vesca*, AMR72055.1), PdHsfA7a (*Prunus dulcis*, XP_034227467.1), PpHsfA7a (*Prunus persica*, XP_020425668.1), PaHsfA6b-like (*Prunus avium*, XP_021821964.1) and MsHsfA6b-like (*Malus sylvestris*, XP_050109366.1), ZjHsfA7a (*Ziziphus jujuba*, XP_015891434.2), DzHsfA7a-like (*Durio zibethinus*, XP_022759739.1), and HtHsfA6B (*Hibiscus trionum*, GMI73800.1). The Hsf protein sequence of *A. thaliana* was obtained from the TAIR database (https://www.arabidopsis.org/) (accessed on 23 March 2025). Then, phylogenetic trees with 1000 bootstrap replicates were created in MEGA 11 using the neighbor-joining (NJ) method [35]. Multiple sequence alignment and visualization were performed using BioEdit and ClustalX 1.81 software [36,37]. Protein properties, including the relative molecular mass, isoelectric point, instability index, and average hydropathicity, of RhHsfA7 were analyzed by using the Expasy-ProtParam tool (http://web.expasy.org/protparam/) (accessed on 23 March 2025) [38]. The signal peptide and transmembrane structures of RhHsfA7 were, respectively, predicted by the online tools SignalP-5.0 (https://services.healthtech.dtu.dk/services/SignalP-5.0/) (accessed on 23 March 2025) and TMHMM-2.0 (https://services.healthtech.dtu.dk/services/TMHMM-2.0/) (accessed on 23 March 2025). The secondary and tertiary structures of the RhHsfA7 protein were, respectively, analyzed on the websites SOPMA (https://npsa.lyon.inserm.fr/cgi-bin/npsa_automat.pl?page=/NPSA/npsa_sopma.html) (accessed on 23 March 2025) and SWISS-MODEL (https://swissmodel.expasy.org/) (accessed on 23 March 2025).

### 4.3. Subcellular Localization of RhHsfA7

The full-length CDS of *RhHsfA7* was cloned into the pSuper1300 vector (China agricultural university, Beijing, China) using the primers listed in Supplementary Materials Table S1. The cloning process involved restriction enzymes HandIII and KpnI. Subsequently, both the *Super*:RhHsfA7-GFP recombinant plasmid and the empty vector (EV) (serving as a control) were transformed into *Agrobacterium tumefaciens* EHA105. This strain was then used for infiltrating young leaves of *Nicotiana benthamiana* plants. To observe the expression and localization of *Super*:RhHsfA7-GFP, a confocal microscope (Olympus FV3000, Tokyo, Japan) was employed to visualize the fluorescence signal two days post-infiltration.

### 4.4. Transcription Activation Assay

The full-length CDS of *RhHsfA7* was amplified using gene-specific primers (Supplementary Materials Table S1) and subsequently cloned into the pGBKT7 vector. The recombinant plasmid pGBKT7-RhHsfA7 (BD-RhHsfA7), along with the positive control (BD-VP16) and the empty vector (BD), was transformed into the yeast strain Y2HGold (Coolaber, Beijing, China). Transformed yeast cells were cultured on an SD/-Trp medium. Serial tenfold dilutions (10^0^, 10^−1^, 10^−2^, and 10^−3^) of yeast cultures were prepared and spotted onto SD/-Trp-His and SD/-Trp plates, respectively. After incubation at 29 °C for 3 days, the yeast cells were photographed.

### 4.5. Quantitative Real-Time PCR Analyses

Total RNAs were extracted from *R. hybrida* materials and then reverse-transcribed into cDNA using the SPARKscipt II All-in-one RT SuperMix for qPCR (SparkJade Biotech, Shandong, China). qRT-PCR was performed using the 2×RealStar Fast SYBR qPCR Mix (GenStar, Beijing, China) on the Bio-Rad CFX Opus 96 Real-Time PCR System (Bio-Rad, Singapore). The *RhUBI2* gene served as the internal control [39], and the expression levels of the *RhHsfA7* gene were calculated by the 2^−∆∆CT^ method [40]. The qRT-PCR primers used are summarized in Supplementary Materials Table S1.

### 4.6. VIGS and Transient Overexpression of RhHsfA7 in Rose Leaves

The VIGS approach [26] was utilized to silence *RhHsfA7* in rose leaves. A 361 bp fragment of the 3′ region of *RhHsfA7* was cloned into pTRV2, generating the pTRV2-*RhHsfA7* construct. pTRV2-*RhHsfA7*, pTRV2, and pTRV1 were separately transformed into *Agrobacterium tumefaciens* EHA105. The transformed bacteria were cultured in an LB medium containing 50 mg/L kanamycin and 25 mg/L rifampicin. The cells were centrifuged (7000 rpm for 8 min) following overnight incubation (28 °C, 180 rpm) and re-suspended in an infiltration buffer (10 mM MgCl_2_, 200 μM AS, and 10 mM MES, pH 5.6) to an OD_600_ of 1.6. Cells containing pTRV1 were mixed with those containing pTRV2-*RhHsfA7* or pTRV2 in a 1:1 (*v*/*v*) ratio and incubated in the dark for 4 h. Fresh, green, and uniformly sized tender leaves from the same position of the roses were infiltrated with strains harboring TRV-*RhHsfA7* and TRV (negative control) under vacuum at 0.8 atmospheres, and then washed with deionized water. The leaves were placed on a water agar medium and incubated in darkness at 24 °C for 2 d, and then subjected to heat stress at 42 °C for 12 h. After heat treatment, the leaves were maintained at 24 °C under a 16 h light/8 h dark cycle for phenotypic observation.

For transient overexpression of *RhHsfA7*, the *A. tumefaciens* EHA105 carrying pSuper1300 (EV) or *RhHsfA7*-OE was collected by centrifugation, and re-suspended in the infiltration buffer to a final OD_600_ of 0.8. The infiltration procedure was performed similarly to in the VIGS experiment.

Leaf phenotypes were documented using a digital camera, and gene expression analyses were conducted.

### 4.7. Statistical Analyses

Statistical analyses were carried out using GraphPad Prism v9. Data were compared using Student’s *t*-tests (ns *p* > 0.05, * *p* ≤ 0.05, ** *p* ≤ 0.01, *** *p* ≤ 0.001, **** *p* ≤ 0.0001) or one-way analyses of variance with a significance level of 0.05. The experimental statistics are reported as means ± standard deviations (SDs).

## 5. Conclusions

In this study, an Hsf gene, *RhHsfA7*, was isolated from *R. hybrida* ‘Samantha’. The full-length CDS of *RhHsfA7* spans 1086 bp, encoding 361 amino acids. The RhHsfA7 protein exhibits a molecular weight of 41.21 kDa and an isoelectric point of 5.41, with no signal peptide or transmembrane structure. Phylogenetic analysis shows that *RhHsfA7* is most closely related to *AtHsfA7a*, *AtHsfA7b*, and *AtHsfA6b* in *Arabidopsis,* and is more closely aligned with Rosaceae species than with other species. The RhHsfA7 protein possesses conserved OD, DBD, NLS, and NES domains, along with the AHA domain, which is specific to the HsfA subfamily. Structural analysis revealed abundant alpha helices and random coils. RhHsfA7 was localized in the nucleus and showed transcriptional activation activity. Expression analysis revealed that *RhHsfA7* is expressed at higher levels in roots and leaves compared to other tissues. *RhHsfA7* was specifically induced by heat stress, but not by salt stress. Functional assays showed that silencing *RhHsfA7* in rose leaves enhanced heat sensitivity, while overexpressing *RhHsfA7* improved heat tolerance. In conclusion, our findings demonstrate that RhHsfA7 positively regulates heat tolerance in roses.

## Data Availability

All data generated or analyzed during this study are included in this published article.

## Supplementary Materials

The following supporting information can be downloaded at: https://www.mdpi.com/article/10.3390/plants14081155/s1. Table S1. The primers sequences used in this study.

## References

[1] Raymond O., Gouzy J., Just J., Badouin H., Verdenaud M., Lemainque A., Vergne P., Moja S., Choisne N., Pont C. (2018). The Rosa genome provides new insights into the domestication of modern roses. Nat. Genet..

[2] Chen C., Hussain N., Wang Y., Li M., Liu L., Qin M., Ma N., Gao J., Sun X. (2020). An Ethylene-inhibited NF-YC Transcription Factor RhNF-YC9 Regulates Petal Expansion in Rose. Hortic. Plant J..

[3] Qi W., Zhang C., Wang W., Cao Z., Li S., Li H., Zhu W., Huang Y., Bao M., He Y. (2021). Comparative transcriptome analysis of different heat stress responses between self-root grafting line and heterogeneous grafting line in rose. Hortic. Plant J..

[4] Zaccai M., Ackerman R., Genis O., Riov J., Zik M. (2009). The bent peduncle phenomenon in roses is a developmental process involving auxin. Plant Sci..

[5] Li Z.Q., Xing W., Luo P., Zhang F.J., Jin X.L., Zhang M.H. (2019). Comparative transcriptome analysis of *Rosa chinensis* ‘Slater’s crimson China’ provides insights into the crucial factors and signaling pathways in heat stress response. Plant Physiol. Biochem..

[6] Qian R., Hu Q., Ma X., Zhang X., Ye Y., Liu H., Gao H., Zheng J. (2022). Comparative transcriptome analysis of heat stress responses of *Clematis lanuginosa* and *Clematis crassifolia*. BMC Plant Biol..

[7] Kumar A., Changwal C., Thapa B., Tanpure R.S., Hada A., Singh P.K., Ghuge S.A. (2021). Transcription factors: A tool box for countering the effect of abiotic stresses. Stress Tolerance in Horticultural Crops.

[8] Yan Y., Zhao J., Lin S., Li M., Liu J., Raymond O., Vergne P., Kong W., Wu Q., Zhang X. (2023). Light-mediated anthocyanin biosynthesis in rose petals involves a balanced regulatory module comprising transcription factors RhHY5, RhMYB114a, and RhMYB3b. J. Exp. Bot..

[9] Fan K., Mao Z., Ye F., Pan X., Li Z., Lin W., Zhang Y., Huang J., Lin W. (2021). Genome-wide identification and molecular evolution analysis of the heat shock transcription factor (HSF) gene family in four diploid and two allopolyploid *Gossypium* species. Genomics.

[10] Guo M., Liu J., Ma X., Luo D., Gong Z., Lu M. (2016). The plant heat stress transcription factors (HSFs): Structure, regulation, and function in response to abiotic stresses. Front. Plant Sci..

[11] Jacob P., Hirt H., Bendahmane A. (2017). The heat-shock protein/chaperone network and multiple stress resistance. Plant Biotechnol. J..

[12] Chen S., Jiang J., Han X., Zhang Y., Zhuo R. (2018). Identification, expression analysis of the hsf family, and characterization of class A4 in *Sedum Alfredii* hance under cadmium stress. Int. J. Mol. Sci..

[13] Scharf K.D., Berberich T., Ebersberger I., Nover L. (2012). The plant heat stress transcription factor (hsf) family: Structure, function and evolution. Biochim. Biophys. Acta.

[14] Huang Y.-C., Niu C.-Y., Yang C.-R., Jinn T.-L. (2016). The heat-stress factor *HSFA6b* connects ABA signaling and ABA-mediated heat responses. Plant Physiol..

[15] Larkindale J., Hall J.D., Knight M.R., Vierling E. (2005). Heat Stress Phenotypes of *Arabidopsis* Mutants Implicate Multiple Signaling Pathways in the Acquisition of Thermotolerance. Plant Physiol..

[16] Zang D., Wang J., Zhang X., Liu Z., Wang Y. (2019). *Arabidopsis* heat shock transcription factor HSFA7b positively mediates salt stress tolerance by binding to an E-box-like motif to regulate gene expression. J. Exp. Bot..

[17] Mesihovic A., Ullrich S., Rosenkranz R.R., Gebhardt P., Bublak D., Eich H., Weber D., Berberich T., Scharf K.-D., Schleiff E. (2022). *HsfA7* coordinates the transition from mild to strong heat stress response by controlling the activity of the master regulator *HsfA1a* in tomato. Cell Rep..

[18] Li M., Zhang R., Zhou J., Du J., Li X., Zhang Y., Chen Q., Wang Y., Lin Y., Zhang Y. (2023). Comprehensive analysis of HSF genes from celery (*Apium graveolens* L.) and functional characterization of *AgHSFa6-1* in response to heat stress. Front. Plant Sci..

[19] Mou S., He W., Jiang H., Meng Q., Zhang T., Liu Z., Qiu A., He S. (2024). Transcription factor CaHDZ15 promotes pepper basal thermotolerance by activating *HEAT SHOCK FACTORA6a*. Plant Physiol..

[20] Xin H., Zhang H., Chen L., Li X., Lian Q., Yuan X., Hu X., Cao L., He X., Yi M. (2010). Cloning and characterization of *HsfA2* from Lily (*Lilium longiflorum*). Plant Cell Rep..

[21] Wang Y., Song C., Tong S., Guo Y., Yang X., Li C., Shao Y., Yi M., He J. (2024). The diversity in interaction between HsfA2 and ACTIN leads to differences in heat stress responses among different lily varieties. Ornam. Plant Res..

[22] Zhang X., Xu W., Ni D., Wang M., Guo G. (2020). Genome-wide characterization of tea plant (*Camellia sinensis*) Hsf transcription factor family and role of *CsHsfA2* in heat tolerance. BMC Plant Biol..

[23] Ding M., Xing W., Li Z., Jin X., Yu Q., Sun J. (2024). The class B heat shock factor *RcHsf17* from *Rosa chinensis* enhances basal thermotolerance in *Rosa rugosa*. Environ. Exp. Bot..

[24] Kang Y., Sun P., Yang Y., Li M., Wang H., Sun X., Jin W. (2024). Genome-Wide Analysis of the Hsf Gene Family in *Rosa chinensis* and *RcHsf17* Function in Thermotolerance. Int. J. Mol. Sci..

[25] Li S., Sun Y., Hu Z., Dong F., Zhu J., Cao M., Wang C. (2025). Cloning and expression analysis of *RhHsf24* gene in Rose (*Rosa hybrida*). Sci. Rep..

[26] Tian J., Pei H., Zhang S., Chen J., Chen W., Yang R., Meng Y., You J., Gao J., Ma N. (2014). TRV–GFP: A modified *Tobacco rattle virus* vector for efficient and visualizable analysis of gene function. J. Exp. Bot..

[27] Huang H., Chang K., Wu S. (2018). High irradiance sensitive phenotype of *Arabidopsis hit2/xpo1a* mutant is caused in part by nuclear confinement of AtHsfA4a. Biol. Plant..

[28] Fragkostefanakis S., Röth S., Schleiff E., Scharf K. (2015). Prospects of engineering thermotolerance in crops through modulation of heat stress transcription factor and heat shock protein networks. Plant Cell Environ..

[29] Maheswari U., Jabbari K., Petit J.-L., Porcel B.M., E Allen A., Cadoret J.-P., De Martino A., Heijde M., Kaas R., La Roche J. (2010). Digital expression profiling of novel diatom transcripts provides insight into their biological functions. Genome Biol..

[30] Qian G., Meng X., Wang S., Mi Y., Qin Z., Liu T., Zhang Y., Wan H., Chen W., Sun W. (2023). Genome-wide identification of HSF gene family and their expression analysis in vegetative tissue of young seedlings of hemp under different light treatments. Ind. Crops Prod..

[31] Xu Y., Jin Y., He D., Di H., Liang Y., Xu Y. (2023). A Genome-Wide Analysis and Expression Profile of Heat Shock Transcription Factor (Hsf) Gene Family in *Rhododendron simsii*. Plants.

[32] Hu Y., Han Y.-T., Wei W., Li Y.-J., Zhang K., Gao Y.-R., Zhao F.-L., Feng J.-Y. (2015). Identification, isolation, and expression analysis of heat shock transcription factors in the diploid woodland strawberry *Fragaria vesca*. Front. Plant Sci..

[33] Zhou L., Yu X., Wang D., Li L., Zhou W., Zhang Q., Wang X., Ye S., Wang Z. (2021). Genome-wide identification, classification and expression profile analysis of the HSF gene family in *Hypericum perforatum*. PeerJ.

[34] Rao S., Das J.R., Balyan S., Verma R., Mathur S. (2022). Cultivar-biased regulation of *HSFA7* and *HSFB4a* govern high-temperature tolerance in tomato. Planta.

[35] Tamura K., Stecher G., Kumar S. (2021). MEGA11: Molecular Evolutionary Genetics Analysis Version 11. Mol. Biol. Evol..

[36] Thompson J.D., Gibson T.J., Plewniak F., Jeanmougin F., Higgins D.G. (1997). The CLUSTAL_X windows interface: Flexible strategies for multiple sequence alignment aided by quality analysis tools. Nucleic Acids Res..

[37] Hall T.A. (1999). Bioedit: A user-friendly biological sequence alignment editor and analysis program for windows 95/98/ NT. Nucleic Acids Symp. Ser..

[38] Duvaud S., Gabella C., Lisacek F., Stockinger H., Ioannidis V., Durinx C. (2021). Expasy, the Swiss Bioinformatics Resource Portal, as designed by its users. Nucleic Acids Res..

[39] Zhao Q., Jing W., Fu X., Yang R., Zhu C., Zhao J., Choisy P., Xu T., Ma N., Zhao L. (2024). TSPO-induced degradation of the ethylene receptor RhETR3 promotes salt tolerance in rose (*Rosa hybrida*). Hortic. Res..

[40] Livak K.J., Schmittgen T.D. (2001). Analysis of relative gene expression data using real-time quantitative PCR and the 2^−ΔΔCT^ method. Methods.

