# Supplementary material for: Characterization and Functional Analysis of RhHsfA7, a Heat Stress Transcription Factor in Roses (Rosa hybrid ‘Samantha’)
Source: Plants (Basel). 2025 Apr 8;14(8):1155. doi: 10.3390/plants14081155 (PMC12030547; doi:10.3390/plants14081155)
Supplement: Supplementary file 1 [file plants-14-01155-s001.zip › plants-3575027-supplementary.pdf]

plants

MDPI

Supplementary Materials Table S1. The primers sequences used in this study.

Primer Name	Primer sequence (5'-3')	Usage
RhHsfA7-F	TGAACACCTAACTGGGTGGG	For RhHsfA7 gene cloning
RhHsfA7-R	GGTCACTGAGGCAGTGTACT	
TRV-*RhHsfA7*-F	TGAGTAAGGTTACC*GAATTC*GGAGAAACGGAAGGAGCTTGAAGAA	For construction of TRV-*RhHsfA7* vector
TRV-*RhHsfA7*-R	GTGAGCTCGGTACCGGATCCCTAAGTAACCTAAGCGATCAGCCAA	
BD-RhHsfA7-F	TGGCCATGGAGGCCGAA*TTC*ATGAATTACTTGTACCCAGT	For construction of pGBKT7-RhHsfA7 vector
BD-RhHsfA7-R	CGCTGCAGGTCGACGGATCCCTAATTTGGGCTTGAACCTA	
RhHsfA7-OE-F	CAAATCGACTCTAG*TCTAGA*ATGAATTACTTGTACCCAGT	For construction of *Super*:RhHsfA7-GFP vector
RhHsfA7-OE-R	CCCTTGCTCACCATGGTACCATTTGGGCTTGAACCTAAGT	
qRT-*RhHsfA7*-F	TTGGGATGAACTATTTAGCGAGAGG	For qRT-PCR of *RhHsfA7*
qRT-*RhHsfA7*-R	CTAAGTAACCTAAGCGATCAGCC	
qRT-*RhUBI2*-F	GCCCTGGTGCGTTCCCAACTG	For qRT-PCR of *RhUBI* (Control)
qRT-*RhUBI2*-R	CCTGCGTGTCTGTCCGCATTG	
